# A Self‐Healing Crease‐Free Supramolecular All‐Polymer Supercapacitor

**DOI:** 10.1002/advs.202100072

**Published:** 2021-05-01

**Authors:** Funian Mo, Qing Li, Guojin Liang, Yuwei Zhao, Donghong Wang, Yan Huang, Jun Wei, Chunyi Zhi

**Affiliations:** ^1^ Flexible Printed Electronics Technology Center Harbin Institute of Technology Shenzhen Nanshan District Shenzhen Guangdong Province 518055 China; ^2^ Department of Materials Science and Engineering City University of Hong Kong 83 Dachi Road Kowloon Hong Kong SAR 999077 China

**Keywords:** all‐polymer approach, crease‐free, self‐healing capabilities, supercapacitors, supramolecular hydrogels

## Abstract

While traditional three‐layer structure supercapacitors are under mechanical manipulations, the high‐stress region concentrates, inevitably causing persistent structural problems including interlayer slippage, crease formation, and delamination of the electrode–electrolyte interface. Toward this, an all‐polymeric, all‐elastic and non‐laminated supercapacitor with high mechanical reliability and excellent electrochemical performance is developed. Specifically, a polypyrrole electrode layer is in situ integrated into a silk fibroin‐based elastic supramolecular hydrogel film with extensive hydrogen and covalent bonds, where a non‐laminate device is realized with structural elasticity at the device level. The non‐laminate configuration can avoid slippage and delamination, while the elasticity can preclude crease formation. Furthermore, under more severe mechanical damage, the supercapacitors can restore the electrochemical performance through non‐autonomous self‐healing capabilities, where the supramolecular design of host–guest interactions in the hydrogel matrix results in a superior self‐healing efficiency approaching ≈95.8% even after 30 cutting/healing cycles. The all‐elastic supercapacitor delivers an areal capacitance of 0.37 F cm^−2^ and a volumetric energy density of 0.082 mW h cm^−3^, which can well‐maintain the specific capacitance even at −20 °C with over 85.2% retention after five cut/healing cycles.

## Graphical abstract

An integrated all polymer‐based supercapacitor with superior reliability and durability is developed, which exhibits a self‐healing efficiency approaching 100% via supramolecular chemistry of host–guest interactions between *β*‐cyclodextrin and amino acid side chains of silk fibroin.

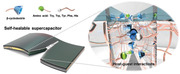

## 1 Introduction

The fast development of flexible and wearable electronics creates a growing demand of energy storage devices with strict performance requirements, especially to meet inevitable mechanical manipulations,^[^ ^1^, ^2^, ^3^ ^]^ where device mechanical reliability weighs more precedence compared to energy storage performance in realistic applications, since the irreversibly structural deformations and damages can result in deadly device performance. However, regarding the mechanical properties and reliability of an integrated flexible device, researches are dominantly focused on individual component layer, while detailed investigations upon structural variation at device level are limited and generally overlooked.

**Figure 1:**
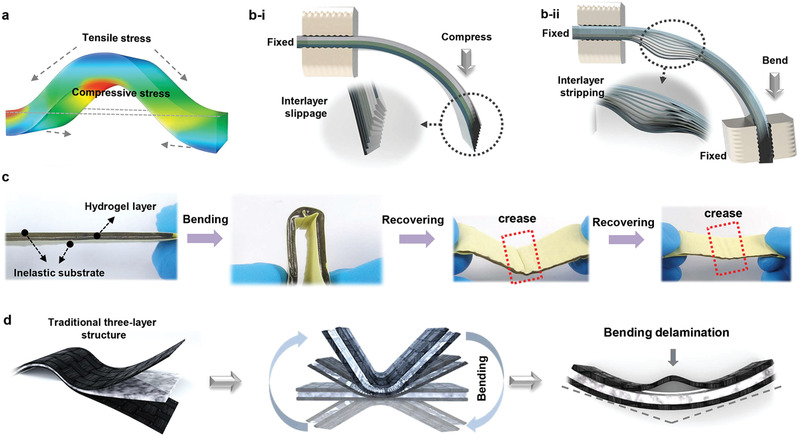
a) Stress distribution model of a device under bending deformation. Schematic illustration of the multilayered structure device under various bending deformations of (b[i]) one end fixed bending and (b[ii]) two‐end fixed bending. c) Schematic illustration of the traditional three‐layer structural device with inelastic electrode substrate upon bending deformation. d) Schematic illustration of the traditional three‐layer structure under repeated bending deformation.

Traditional flexible energy storages normally possess a laminated multilayer configuration consisting of a polymer electrolyte sandwiched between two stacked electrodes.^[^ ^4^ ^]^ In order to delineate the possible structural damages, at beginning, it is necessary to carefully analyze the device structural evolution under mechanical manipulations, and the resulting device failure can be mainly reflected from aspects of device level and electrode level. Taking the most general bending deformations with centripetal load as examples, the high‐stress region will concentrate near around the maximum strain corner of device with both tensile and compressive stresses (**Figure** **1a**). Specifically, when the multilayered structure device with one end fixed is bent repeatedly for a couple of times, the interlayer slippage can occur between contiguous layers (Figure 1b [i]). While the device is clamped at both ends and then bent, the interlayers can strip at the strain corner (Figure 1b [ii]). In these conditions, the original structure integration at device level would be damaged when the strain exceeds the withstanding mechanical limits and cause irreversible layer delamination. On the other hand, after multiple bending deformation of a modeled three‐layered device (Figure 1c), the inelastic electrode layers, sandwiching the elastic hydrogel electrolyte layer, would result in interlayer stripping. Even when the stress is removed, the device cannot be fully recovered, and the crease will be permanently stay behind for the electrode layer. Besides, after experiencing substantial bending deformation, delamination normally appears in the in‐plane bending corner of a sandwiched structural device (Figure 1d). Therefore, bending manipulations can cause the interlayer slippage and irreversible layer delamination for the whole device due to the weak interfacial binding between electrode and electrolyte layers, and the crease phenomenon due to inelastic characteristic of electrode layers. Undoubtedly, these structural instabilities can spark off device performance deterioration and even dysfunctionality under realistic service.

It imposes great challenges for device configuration, modification, and material design to resolve the aforementioned structural issues. First, to avoid interfacial delamination, the most effective strategy is to introduce chemical bonds at the interfacial spots to replace the general hydrogen bonds and/or Van der Waals force. If two contiguous layers encompass the same networks penetrating into each other, an all‐in‐one configuration can be consequently developed without obvious interface existing, which avoids laminate configurations. Second, to avoid the crease phenomenon, it requires elasticity of both electrode layer and the electrolyte layer to dissipate the loading force. To embody the proposed strategies, we build a model with all‐in‐one configuration and all‐elastic layers based on sponge, which can fully resile after arbitrary deformations (Figure S1, Supporting Information). When under bending, the outer layer is under tensile stress, while the inner layer is under compression, this will cause the crease formation at the inner layer. After stress is unloaded, the crease will be recovered and return to a smooth surface. According to these analyses, therefore, the key to the abovementioned problems is the certain elasticity of both the electrode and electrolyte layers.

Furthermore, compared to the abovementioned general mechanical manipulations, the flexible and wearable energy storages in practical applications have large chance to encounter even severer conditions, such as deadly breaking and cutting, resulting in the end of device lifetime. Fortunately, self‐healing science has been considered as a promising strategy that the self‐healable supercapacitors can spontaneously restore their mechanical structure and capacitance performances after suffering from physical damages or abuses.^[^ ^5^, ^6^, ^7^ ^]^ Substantial efforts have been dedicated to developing intrinsically autonomic self‐healing supercapacitors fabricated by various healable polyelectrolyte (e.g., hydrogen bonding, metal ionic bonding, *π*–*π* conjugation, or ester bonding).^[^ ^8^, ^9^, ^10^, ^11^ ^]^ However, among these achievements of noncovalent bonding‐aided healing mechanism, the maximum breaking/healing cycles is only 20,^[^ ^11^ ^]^ which is still unsatisfactory to meet the needs of diverse practical scenarios. In addition, the broken electrodes in most traditional self‐healing supercapacitors were generally not intrinsically self‐healable, which normally need extra conductive patches and outer healable layer, leading to the operation difficulties and decrease of efficiency. On the other side, little emphasis has been placed on the multifunctional hydrogel electrolytes that possessing both intrinsic self‐healing and freeze‐resistant properties up to now. While working in subzero temperatures, the aqueous solutes of hydrogel electrolytes tend to freeze, which dramatically deteriorate the device reliability involving self‐healability, flexibility, and the ionic conductivity of the electrolytes. Increasing the salt content can effectively improve the freeze‐resistance of the hydrogel electrolyte. However, due to the salt‐out effect, such high‐concentration salt will inevitably affect the mobility of hydrogel network which is vital for self‐healing process.^[^ ^12^ ^]^

In this study, we fabricated a highly reliable supercapacitor based on the supramolecular hydrogel (supramolecular supercapacitor) through an all‐elastic and all polymer approach, in which all the components have been integrated into a single supramolecular hydrogel film, differing from the laminated multilayer configuration of a traditional supercapacitor. The supramolecular hydrogel contained two kinds of *β*‐cyclodextrin (*β*‐CD)‐based host–guest inclusion complexes with superior self‐healing properties. *β*‐CD was conjugated onto silk fibroin and polyacrylic acid (PAA) molecular chains via Schiff base formation,^[^ ^13^ ^]^ and stable inclusion complexes were formed by host–guest interactions between the grafted *β*‐CD and the neighboring amino acid molecules on silk fibroin to construct the supramolecular hydrogel. Furthermore, polypyrrole (PPy) was integrated into the supramolecular hydrogel electrolyte through extensive hydrogen bonding and covalent interactions with aromatic amino acid residues of silk fibroin chains to serve as the elastic electrodes, which exhibited high areal capacitance and volumetric energy density. With this unique structure design, the all‐in‐one integrated supercapacitor exhibited superior mechanical reliability to survive from general mechanical manipulations to severe damages, low‐temperature resistance, and self‐healability with high healing efficiency, all which contributes a more reliable and durable supercapacitor.

## 2 Formulation of All‐Polymer Elastic Non‐Laminate Structured Supercapacitor

The free‐standing supramolecular hydrogel electrolyte is synthesized by photoinitiated radical polymerization of PAA‐*β‐*CD and supramolecular self‐assembly of host–guest molecule conjugated chains (Figure S2, Supporting Information). Host–guest interaction is an efficient quasi‐covalent interaction that combines multiple dynamic interactions.^[^ ^14^, ^15^, ^16^ ^]^ In general, intrinsic hydrophobic internal cavity of the cyclodextrins can encapsulate diverse guest molecules to form physical inclusion complex via supramolecular bonds, which endows superior self‐healing property in supramolecular hydrogels. As guest molecules, a large variety of aromatic amino acid residues (e.g., tyrosine [Tyr], tryptophan [Trp], phenylalanine [Phe], and histidine [His]) in peptides of silk fibroin chains can be applied in the preparation of self‐healing hydrogels.^[^ ^17^ ^]^ Comparing with other self‐healing mechanisms, such as hydrogen bonding, metal ionic bonding, *π*–*π* conjugation, and ester bonding (Figure S3, Supporting Information), host–guest interaction is a more efficient noncovalent interaction that is closer to covalent interaction, thus it can be hypothesized that the healing efficiency of the designed supramolecular hydrogels would be highly improved.

**Figure 2:**
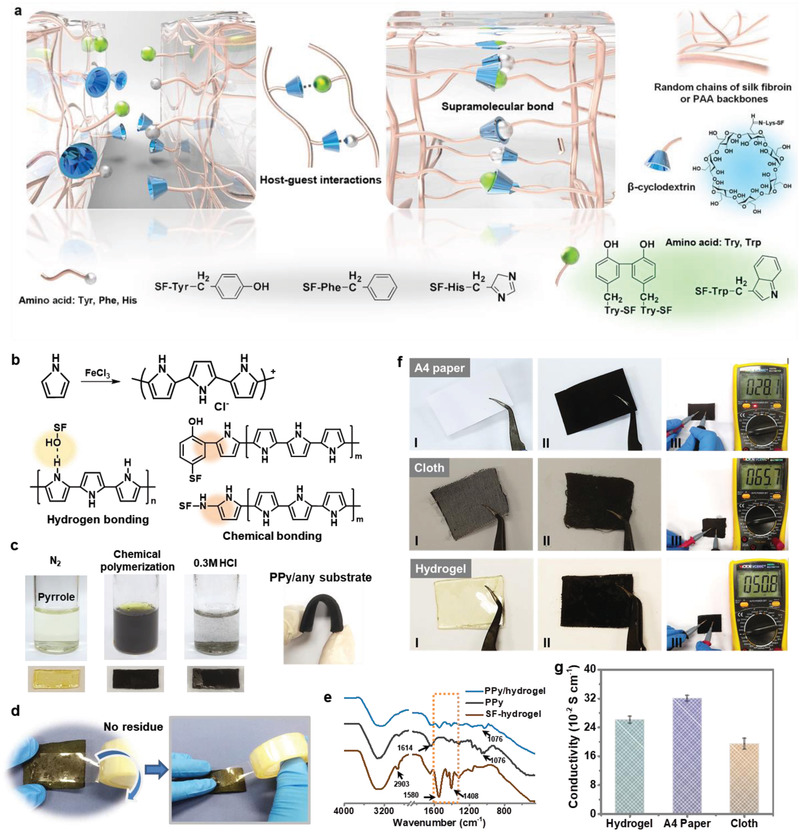
a) Schematic diagram of the self‐healing mechanism of the supramolecular hydrogels. Supramolecular bonds were formed through host–guest interactions between the grafted *β*‐CD molecules and residual amino acid molecules on the interactive surfaces of the supramolecular hydrogel pieces. b) Chemical reaction schemes of the polymerization of Py, and the interactions between PPy and amino acid side chains of silk fibroin including Ser, Thr, Lys, and Tyr. c) Digital photographs of the supramolecular hydrogel during polymerization process. d) The tape test of PPy‐coated supramolecular hydrogel shows the strong mechanical property. e) FTIR spectra of the PPy‐coated supramolecular hydrogel. Digital photographs of various polymer materials f[I] in pristine condition, f[II] after treament, and f[III] under electricity resistance measurement. g) Electrical conductivity of various polymer materials after electronic modification.

The fabricated supramolecular hydrogel consisted of a homogeneous dual network structure, in which the *β*‐CD conjugated silk fibroin chains interact with conjugated PAA chains through physical intertwining and supramolecular bonds, as illustrated in **Figure** **2a**. For the first network, the *β*‐CD monoaldehyde was synthesized and conjugated onto silk fibroin chains with lysine amines (Lys) through Schiff base formation. The other network of *β*‐CD conjugated PAA was constructed by chemically cross‐linked the *β*‐CD grafted acrylic acid monomer through free‐radical polymerization. Owing to the fact that Tyr, Trp, Phe, and His residual amino acid were highly suited for host−guest interactions with *β*‐CD,^[^ ^18^ ^]^ the grafted *β*‐CD between the two networks binds with the neighboring amino acid on the silk fibroin side chains, thus supramolecular bonding interactions were formed in the dual‐cross‐linked hydrogels (Figure S4, Supporting Information). According to the dynamic characteristics of quasi‐covalent cross‐linking sites of the host–guest interactions between extensive *β*‐CD and amino acids, it can be hypothesized that the resultant supramolecular hydrogels would achieve efficient self‐healing ability, which can autonomously repair themselves without external stimuli (Figure 2a).

The next step is to fabricate the polymeric elastic electrodes. Owing to its high conductivity, remarkable pseudocapacitance, noncytotoxicity, and intrinsic flexibility, conductive PPy has received widespread attention as typical polymer electrode material of supercapacitors.^[^ ^19^, ^20^, ^21^ ^]^ In addition, polymerized PPy molecules can form extensive hydrogen bonding and covalent interactions with aromatic amino acid residues (Lys and Tyr) of silk fibroin chains (Figure 2b), thus be fixed instead of simply adsorbed by the gel matrix, showing a good compatibility to the supramolecular hydrogel.^[^ ^22^ ^]^ The fabrication process of the PPy integrated supramolecular hydrogel electrolyte (PPy/supramolecular hydrogel) is illustrated in Figure 2c. A piece of supramolecular hydrogel was first soaked in pyrrole solution for 20 min, and subsequently immersed into ferric chloride and hydrochloric acid mixed solution to polymerize PPy onto the hydrogel substrate. The supramolecular hydrogel film gradually blackened after 5 min, indicating the beginning of polymerization. The longer polymerization time led to the increase of PPy mass loadings and thereby enhanced the electrical conductivity of PPy/supramolecular hydrogel (Figure S5, Supporting Information). The mechanical adhesion between the PPy and supramolecular hydrogel was also tested by tape. It could be observed that no obvious residual PPy was adhered on the tape after peeling, which indicated the PPy has been diffused into the gel matrix and resulted in a diffuse layer of PPy/hydrogel between the PPy electrode and hydrogel electrolyte (Figure 2d and Figure S6, Supporting Information). The excellent adhesion of PPy on supramolecular hydrogel is essential for electrochemical and mechanical stability of the assembled energy storage devices. The chemical compositions of supramolecular hydrogel before/after PPy coating process were characterized by Fourier transform infrared spectroscopy. As shown in Figure 2e, the spectrum of PPy/supramolecular hydrogel presents almost all the characteristic peaks of PPy, revealing that supramolecular hydrogel was covered by PPy. Additionally, the decreased characteristic peak located at round 2903 cm^−1^ was assigned to the stretching vibration of C—H originated from the pyranoid ring of PPy, indicating that PPy bound to supramolecular hydrogel matrix via the interaction between the N of pyrrole rings and hydroxyl groups of silk fibroin by hydrogen bonding.^[^ ^23^ ^]^ The signals at 1408 and 1580 cm^−1^ were attributed to amino groups of silk fibroin. The decrease peak density of these two signs indicated that the polymerized PPy molecules were fixed onto the silk fibroin hydrogels through the formation of covalent interactions with amino acid residues of silk fibroin chains.

Furthermore, the fabrication process of highly elastic PPy electrode for supercapacitors is highly versatile and suitable for diverse polymer substrate, such as A4 paper and textile (Figure 2f), and the modified substrates all exhibited excellent electrical conductivity (Figure 2g). These results indicated that by using this coating method, PPy could uniformly diffuse and form sufficient interconnected conductive paths in diverse polymeric networks, thus endowing the substrates with excellent electrical conductivity. Moreover, owing to the electrode–electrolyte integrated design, the PPy/supramolecular hydrogel also displayed self‐healing capability by autonomously restoring its electrical conductivity after mechanical damage.

## 3 Electrochemical Performance of the Elastic All‐Polymer Supercapacitor

**Figure 3:**
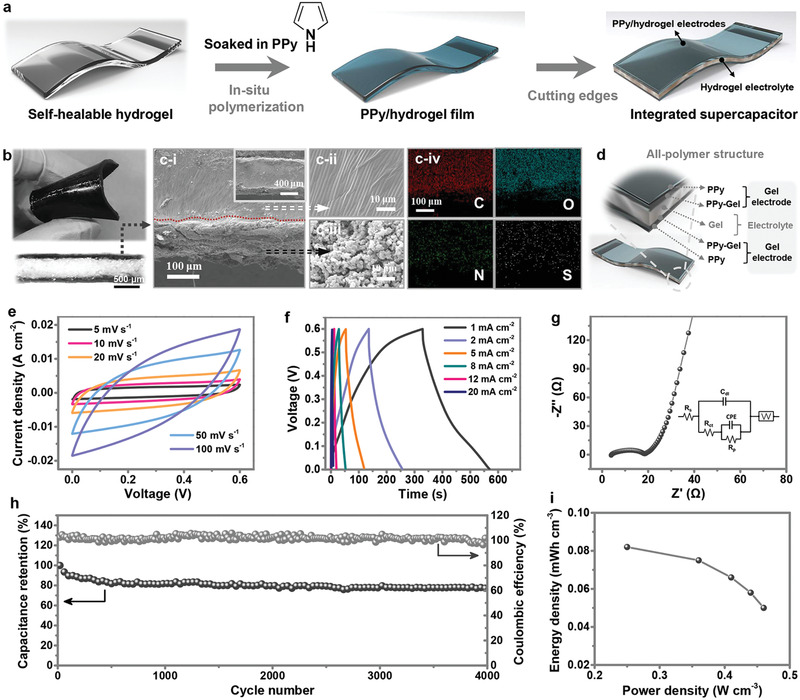
Characterization of the integrated supramolecular supercapacitor. a) Fabrication route of the supramolecular supercapacitor. b) A digital photo and a cross‐sectional microscopy image of the supramolecular supercapacitor. c[i]) Cross‐sectional morphology of the device and the c[ii], c[iii]) enlarged SEM images. c[iv]) The EDS element mapping images which are corresponding with the cross‐sectional morphology (c[i]). d) Schematic of the configuration the supramolecular supercapacitor. e) CV curves at various scan rates. f) GCD curves at various charging/discharging currents. g) Electrochemical impedance spectrum. Inset shows the equivalent circuit. h) Cycle performance tested at 0.5 mA cm^−2^. i) Energy and power densities of the supramolecular supercapacitor.

To fabricate the instantaneous all‐polymer elastic supercapacitor, conducting polymer PPy was first in situ polymerized and bounded to supramolecular hydrogel matrix through extensive hydrogen bonding and covalent interactions with aromatic amino acid residues (Lys and Tyr) of silk fibroin chains as elastic electrodes to finally obtain the all‐elastic and all polymer configuration. Subsequently, a cut along the surrounding edges of the hybrid gel was carried on to avoid short circuit and trim the supercapacitor to a desired dimension (**Figure** **3a**). The as‐obtained supercapacitor was highly flexible and PPy was distributed uniformly onto the surface and subsurface of the supramolecular hydrogel film (Figure 3b). Scanning electron microscope was used to characterize the cross section of the fabricated supramolecular supercapacitor after freeze‐drying. As depicted Figure 3c [i–iii], the PPy network exhibited a cloud‐ or floc‐like appearance, which differed from the relatively flat supramolecular hydrogel layer. Additionally, the element mapping of energy dispersive X‐Ray spectroscopy (EDS) exhibited that nitrogen mainly concentrated on both sides of the supramolecular hydrogel, and this part of nitrogen is originated from PPy network. Meanwhile, small amount of nitrogen uniformly dispersed in gel component, which derived from the residual acid residues of silk fibroin chains (Figure 3c [i–iv]). According to the model of infiltration, it can be hypothesized that the PPy molecules infiltrated from the outer layer of hydrogel electrolyte and formed a PPy–hydrogel composite osmotic layer outside the hydrogel. This osmotic layer is considered as the PPy electrode layer, and the inner supramolecular hydrogel layer is regarded as the electrolyte layer. Due to self‐healing property, this interface is mechanically stable. Thus, the whole configuration of the tightly attached sandwich‐like structure can be proposed as an all‐in‐one structure, which can improve the device reliability (Figure 3d).

In order to achieve better performance of the designed supercapacitor, we first investigated the effects of different concentrations of pyrrole during fabrication. As shown in Figure S7, Supporting Information, galvanostatic charge–discharge (GCD) curves of the supramolecular supercapacitors indicated that the specific capacitance exhibited an increasing trend as the Py concentration increased, and the tendency to change gradually reduced when Py concentration approached 0.5 m. As a proof of concept, the sample with 0.5 m concentration was selected as a model system to execute further electrochemical performance measurements. The detailed capacitive behavior was revealed by the cyclic voltammetry (CV) scans and GCD curves of the constructed supercapacitor with 0.5 m PPy concentration, as shown in Figure 3e,f. The linear profiles of CV curves exhibited quasi‐rectangular mirror symmetry in the potential window of 0–0.6 V, indicating the good capacitive behavior of the supercapacitor. The gradual deviation from the rectangular shape with the increasing scan rate to 100 mV s^−1^ was ascribed to large transfer resistance and limited diffusion at high scan rates. In addition, the specific capacitance of the supercapacitor was determined by the GCD tests performed at various current densities. The resultant GCD curves from 1 to 20 mA cm^−2^ all exhibited typical triangular shape, revealing the excellent charge–discharge reversibility of the device. The areal capacitance versus current density was displayed in Figure S7, Supporting Information, which presented a notable positive correlation with respect to polymerized PPy mass loading. The supercapacitor with 0.5 m PPy concentration delivered the highest areal capacitance of ≈0.37 F cm^−2^ at a current density of 1 mA cm^−2^. This value was much higher than those in previously reported PPy‐based supercapacitors,^[^ ^24^, ^25^, ^26^, ^27^ ^]^ which was ascribed to the in situ polymerized non‐laminated structure favoring the ion diffusion. Besides, volumetric capacitance with respect to the volume of the whole device was calculated to be 556.8 mF cm^−3^ at a current density of 26.3 mA cm^−3^. The good electrochemical performance could be ascribed to the high ionic conductivity of supramolecular hydrogel electrolyte and the enhanced of electronic conduction by non‐laminated electrode–electrolyte design. To characterize the charge transport process, electrochemical impedance spectroscopy (EIS) was performed on the supramolecular supercapacitor, as shown in Figure 3g. The charge‐transfer resistance calculated from the semicircle diameter in the high‐frequency region was as low as 2.7 Ω, indicating a fast kinetic process. Meanwhile, the typical straight sloping line in the low‐frequency region indicated the good capacitive behavior of the supercapacitor. Furthermore, the long‐term cycling stability was investigated by consecutive charging–discharging cycles at the current density of 0.5 mA cm^−2^. The supramolecular supercapacitor exhibited excellent cyclic performance with capacitance retention over 80.5% even after 4000 cycles, indicating its long‐term usability (Figure 3h). In addition, the supramolecular supercapacitor delivered a high volumetric energy density of 0.082 mWh cm^−3^ at a power density of 0.25 W cm^−3^, as well as a high power density of 0.47 W cm^−3^ at an energy density of 0.049 mWh cm^−3^ (Figure 3i, all the values were calculated based on the volume of the whole device). These values are comparable to those of some reported supercapacitor based on ZnO nanocables (0.04 mWh cm^−3^),^[^ ^28^ ^]^ TiN nanowires (0.05 mWh cm^−3^),^[^ ^29^ ^]^ oxidized CNT arrays (0.04 mWh cm^−3^),^[^ ^30^ ^]^ etc.

## 4 Enhanced Mechanical Reliability of the All‐Polymer Elastic Non‐Laminate Structured Supercapacitor

**Figure 4:**
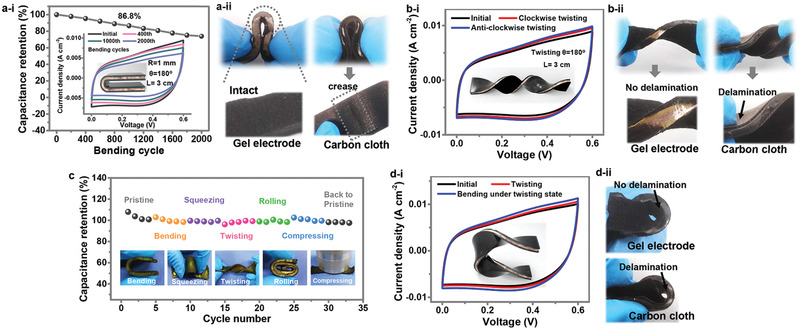
Electrochemical performance of supramolecular supercapacitor under various mechanical stimuli. a[i]) Capacitance retention of the supramolecular supercapacitor at bending test. Inset is the corresponding CV curves recorded at certain cycle intervals. a[ii]) Photographs showing the intact surface of PPy/supramolecular hydrogel, and the clear crease on the surface of the traditional three‐layer supercapacitor with carbon cloth electrodes after bending test. b[i]) CV curves of the supramolecular supercapacitors at 30 mV s^−1^ while it being dynamically bended after twisting deformation. b[ii]) Photographs showing the interfacial contact of the supramolecular supercapacitor and comparative supercapacitor with carbon cloth electrodes under twisting deformation. c) Capacitance retentions of the supramolecular supercapacitor obtained from GCD profiles under various mechanical stimuli. d[i]) CV curves of the supramolecular supercapacitors at 30 mV s^−1^ while it being dynamically bended after twisting deformation. d[ii]) Photographs showing the interfacial contact of the supramolecular supercapacitor and comparative supercapacitor with carbon cloth electrodes under multiple deformations.

Our designed polymer supercapacitor with all‐in‐one structure can be highly resistant to mechanically constant damage due to its all‐elastic feature (**Figure** **4** and Figure S8, Supporting Information). The CV curves almost overlapped as the bending angles increased from 0° to 180°, revealing no decay in capacitance (Figure S9a, Supporting Information). To simulate the repeated mechanical impacts that normally occur in practical use, we employed a specially designed stepper motor to bend the supercapacitor automatically and recorded the capacitance retention at certain cycle intervals (Figure S10, Supporting Information). As shown in Figure 4a [i], during the bending process (L: length of the device, R: bending radius of curvature, *θ*: bending angle), no obvious capacity loss could be detected after bending the supramolecular supercapacitor for 100 times, and nearly 86.8% capacity was retained even after 1000 bending times without appreciable deterioration in CV curves, indicating the good electrochemical stability and mechanical robustness. Besides, further mechanical measurement was performed to investigate the influence of various external forces. The supramolecular supercapacitor exhibited stable electrochemical performance under dynamically twisting state (Figure S9b, Supporting Information). Figure 4a [ii] indicated that the PPy/supramolecular hydrogel exhibited an intact surface after bending test, whereas the comparative traditional three‐layer supercapacitor with carbon cloth as electrodes showed a clear crease on the surface. As expected, when the supercapacitor was in twisting state (L: length of the device, twisting *θ*: the clockwise twisting angle), only subtle changes were detected in the CV curves, indicating a great reliability (Figure 4b [i]). No delamination could be observed in Figure 4b [ii] when the supercapacitor was being twisted, benefiting from the elastic polymeric electrodes and non‐laminated design. As shown in Figure S8c,d, Supporting Information, the EIS results of the all‐elastic supramolecular supercapacitor under different deformation states exhibited that no significant change was detected in the interfacial resistance of the supramolecular supercapacitor during deformations, which further confirmed its stability. For the laminated supercapacitor with carbon cloth electrodes, the change of Warburg impedance at low frequencies was ascribed to the micro changes of the laminated structure of the supercapacitor with carbon cloth electrodes. Furthermore, long‐term cyclic charge/discharge tests were executed under simultaneous mixed mechanical stimuli including bending, squeezing, twisting, rolling, and compressing, as displayed in Figure S11, Supporting Information, and the capacitance retention in each cycle was accordingly calculated from the recorded GCD profiles while being dynamically operated (Figure 4c). The results showed that the corresponding GCD profiles kept smooth without fluctuations during charging/discharging, and the specific capacitance could also be well‐maintained after 33 cycles, furthermore confirming the superior stability and durability of the supramolecular supercapacitor under severe mechanical stimuli. Actually, as the whole device is highly elastic, the stress induced by mechanical stimuli could be largely dissipated while under vast deformations. Moreover, such all‐in‐one structure could eliminate the interfacial resistance between the electrodes and hydrogel electrolyte, therefore, the electrochemical and mechanical stability of the supramolecular supercapacitor was greatly enhanced. When further bending deformation was subsequently applied to the supercapacitor in twisting state, the supramolecular supercapacitor also exhibited a stable electrochemical performance without delamination appearing in the interface between the electrodes and electrolyte, as displayed in Figure 4d. These results indicated that through the device design, material selection, and performance comparison, the mechanical stability and reliability of the devices could be guaranteed even under various severe deformations.

## 5 Self‐Healing of the Supramolecular Supercapacitor

**Figure 5:**
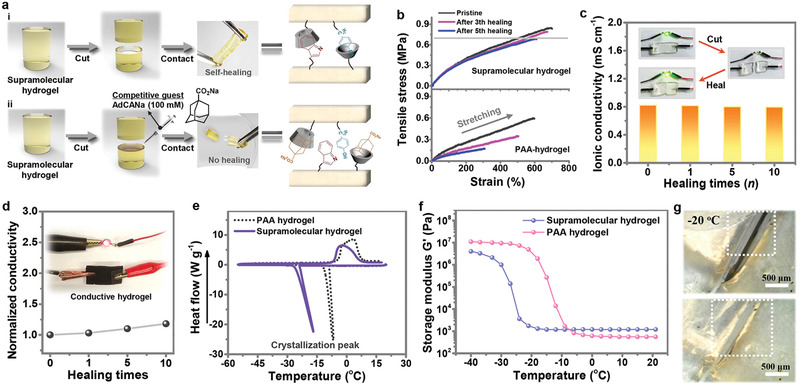
Characteristics of the supramolecular hydrogels. a) Self‐healing experiments of the supramolecular hydrogels via host–guest interactions. When two cut edges of cylindrical hydrogel pieces were contacted together under wet conditions, the pieces healed completely within 1 h (a[i]); If a competitive guest of AdCANa solution was spread on one of the cut surfaces, no healing could be observed a[ii]). b) Tensile strain–stress profiles of the supramolecular hydrogel and the comparative PAA‐hydrogel. c) Ionic conductivity of the supramolecular hydrogel after multiple cutting–healing times. d) Normalized electrical conductivity of the conductive supramolecular hydrogel after multiple breaking/healing cycles. e) DSC curve of the supramolecular hydrogel electrolyte and PAA hydrogel electrolyte at the scan rate of 10 °C min^−1^. f) The storage modulus (G′) of the PAA hydrogel and the supramolecular hydrogel electrolyte. g) Optical microscope images of the self‐healing process of the supramolecular hydrogels while under −20 °C condition.

To demonstrate the self‐healing mechanism of host–guest interaction, adamantane carboxylic acid sodium salt (AdCANa) was utilized as a competitive guest molecule, owing to its higher association constant for *β*‐CD than that of amino acid molecules.^[^ ^16^ ^]^ As illustrated in **Figure** **5a**, a cylindrical‐shaped supramolecular hydrogel was first cut in half, and subsequently the two pieces were rejoined together. After being stored in wet conditions for 1 h, two separated halves healed sufficiently to one holistic gel again with the crack disappeared (Figure 5a [i]). By contrast, when 3 mm competitive guest solution of AdCANa was placed onto the cut surfaces of the supramolecular hydrogel, no healing could be observed after reattaching the two cut pieces for 1 h (Figure 5a [ii]). That is because the competitive AdCANa molecules have filled up the *β*‐CD, thus inhibiting its self‐healing property. This result intuitively indicates that the self‐healing performance of the supramolecular hydrogel is ascribed to the formation of host–guest inclusion complexes between two cut surfaces.

Furthermore, the tensile test quantitatively indicated the large stretchability of the supramolecular hydrogel by delivering a stress of ≈0.8 MPa with a high break elongation over 700%. Remarkably, the tensile stress–strain curves of the healed supramolecular hydrogel after five healing cycles almost overlapped with that of the pristine sample, exhibiting high healing efficiencies of 84.2% and 88.6% calculated from the strength and elongation, respectively (Figure 5b). Owing the existence of hydrogen bonding, PAA hydrogel normally exhibited a certain degree of self‐healing capability^[^ ^31^, ^32^ ^]^ (Figure S12, Supporting Information). However, the mechanical properties of normal PAA hydrogel deteriorate dramatically during cutting–healing process. The supramolecular chemistry of host–guest interactions in the supramolecular hydrogel was a more efficient quasi‐covalent interaction than hydrogen bonding, therefore, supramolecular hydrogel exhibited a better self‐healing efficiency than the PAA hydrogel. These results indicate the excellent self‐healing property of the supramolecular hydrogel. In addition, the ionic conductivity of the supramolecular hydrogel was measured by electrochemical AC impedance spectroscopy test (Figure S13, Supporting Information). The calculated ionic conductivity was almost fully restored to its initial value, even after ten breaking–healing cycles (Figure 5c). The inset showed the reviving process of a light emitting diode (LED) circuit after healing of supramolecular hydrogel. Moreover, the PPy/supramolecular hydrogel also displayed self‐healing capability by autonomously restoring its electrical conductivity after mechanical damage. As displayed in Figure S14, Supporting Information, the cut halves could spontaneously heal to form one gel after contact for 1 h, and the outer healed PPy layer still kept good electrical contact after a couple of breaking/healing cycles, as evidenced in the resistivity measurements (Figure 5d). It could be observed that an LED circuit revived after healing of PPy/supramolecular hydrogel (inset of Figure 5d). In addition, the antifreezing property of the supramolecular hydrogel electrolyte was also investigated systematically, as shown in Figure 5e–g. The differential scanning calorimetry (DSC) result indicated that the freezing point of the supramolecular hydrogel electrolyte was approximately in the range of −22.4 to −25.6 °C, which was much lower than that of the comparative PAA hydrogel electrolyte in the crystallization temperature range of −7.4 to −11.2 °C (Figure 5e). Moreover, no obvious change could be observed in the storage modulus (*G*′) of the supramolecular hydrogel electrolyte when temperature decreased to −20 °C, revealing the stable mechanical flexibility and low‐temperature tolerance of the supramolecular hydrogel electrolyte (Figure 5f). Figure S15, Supporting Information, indicated that the ionic conductivity of supramolecular hydrogel electrolyte was calculated to be 0.82 mS cm^−1^ at room temperature. Upon cooling down to subzero temperatures, the ionic conductivity of supramolecular hydrogel electrolyte decreased as the decrease of diffusion velocity of electrolyte ions at low temperature, but a high ionic conductivity was retained even at −20 °C (0.56 mS cm^−1^), revealing a good low temperature‐resistance of the supramolecular hydrogel electrolyte. Furthermore, while being placed at −20 °C, the hydrogel films could sufficiently heal into the pristine state after 1 h with the crack disappearing as evidenced by optical microscopic observation; this was because that the supramolecular host–guest interaction was not affected by the temperature unlike the hydrogen bonding‐based self‐healing mechanism (Figure 5g). The steadily jointed interface between broken pieces could perfectly keep the integrity after healing process. In addition, the dehydration test results revealed the weight of supramolecular hydrogel remained over 40% weight after 72 h of storage, demonstrating a certain degree of water retention of the supramolecular hydrogel (Figure S16, Supporting Information).

**Figure 6:**
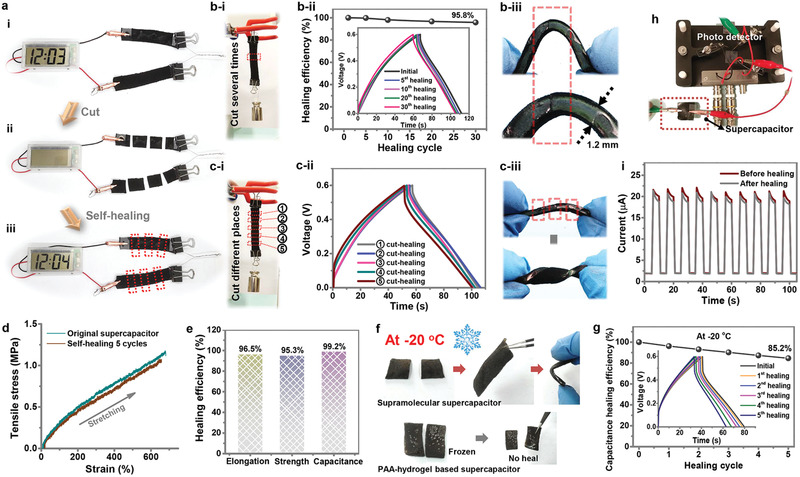
Self‐healing behavior of the integrated supramolecular supercapacitor. Digital photographs of the in‐series supercapacitor circuit to power an electronic watch being under cut/healed condition (a[i] pristine; a[ii] cut; a[iii] healed). b[i]) Demonstration of the self‐healed supercapacitor to support a 500 g mass after clyclic cutting‐healing operation. b[ii]) Electrochemical performance of the healed supercapacitor during cutting–healing cycles. b[iii]) A photograph of the healed supercapacitor under bending deformation with a red rectangle indicating the cyclic wound/healing position. c[i]) Demonstration of the self‐healed supercapacitor to support a 500 g mass after five cutting‐healing process. c[ii]) Electrochemical performance of the healed supercapacitor during five cutting–healing process. c[iii])A photograph of the healed supercapacitor under twisting state with the red rectangles indicating the wound/healing positions). d) Tensile stress–strain curves of original and healed device after five breaking/healing cycles. e) Healing efficiencies of the supercapacitor after five breaking/healing cycles. f) Comparisons of the self‐healing performance of the supramolecular supercapacitor and the PAA‐hydrogel‐based supercapacitor under −20 °C cold condition. g) Capacitance healing efficiency of the supramolecular supercapacitor at −20 °C. h) A photo of the perovskite nanowires‐based photodetector driven by the supercapacitor. i) Performances of the photodetector driven by the original/healed supercapacitor.

The highly self‐healable hydrogel electrolyte promises the mechanical and electrochemical healability of the fabricated supercapacitor. To demonstrate this, two supramolecular supercapacitors was connected in series to power a digital watch, and no difference could be observed when the two supercapacitors were performed for cut/healed operation (**Figure** **6a**). Furthermore, the electrochemical performances of the self‐healable supramolecular supercapacitor before/after healing were systematically investigated. On the first hand, we bisected the supramolecular supercapacitor on the same position for multiple times, and each time the fresh cuts were subsequently rejoined under mild pressure. After standing for 1 h in ambient condition, the breakage was sufficiently self‐healed. As illustrated in Figure 6b, the healed belt‐shaped supercapacitor could be extensively stretched and hoisted a 500 g mass without breaking. Besides, both CV and GCD curves almost overlapped with the capacitance retention rate of the device approaching ≈95.8% (calculated by the GCD profiles) even after the 30 cutting/healing cycles (Figure 6b [i] and Figure S17, Supporting Information). Whereas, the electrochemical performance of the contrastive supercapacitor with AdCANa spread on the cut surface dramatically deteriorated (Figure S18, Supporting Information). These results verify that the superior self‐healing properties via host–guest interactions not only rebuild the mechanical integration but also revive the electrochemical performance at device level. The EIS profile displayed in Figure S19, Supporting Information, revealed the good recovery of electrochemistry characteristics of the supramolecular supercapacitor after the healing process. Furthermore, after the healing process, the supramolecular supercapacitor still exhibited a compatible and tightly attached interface between the electrode and electrolyte without delamination. The steadily jointed interface could perfectly keep the integrity of the whole device even bending the supramolecular supercapacitor to a semicircle (Figure 6b [ii] and Figure S20, Supporting Information). As recorded in Table S1, Supporting Information, the superior healing efficiency of supramolecular supercapacitor extremely outperformed other reported self‐healable devices (54.2–84.7% healing efficiency, no more than 20 cycles), which can be attributed to the highly efficient self‐healable supramolecular hydrogel electrolyte by host–guest interactions and the high electrical conductivity of PPy coating.

On the other hand, the supramolecular supercapacitor was cut on the diverse positions and sequentially tested the electrochemical performance during the five breaking/healing process (Figure 6c [i] and Figure S21, Supporting Information). The slight fluctuation of healing efficiency in GCD curves of Figure 6c could be ascribed to the accidental microcosmic adjustment of the reconnected electrode layers. Similarly, the supramolecular supercapacitor exhibited a tightly attached interface between the electrode and electrolyte in all the cut‐healed position, and could be twisted without the appearance of delamination after healing process (Figure 6c [ii]). These results demonstrated the practical utility of the self‐healable supramolecular hydrogel electrolyte based on host–guest interactions, which offered the assembled supercapacitor with effective self‐healability. In addition, after five breaking/healing cycles, the mechanical properties of the healed devices were well‐restored with a tensile strain of 620%, and decent electrochemical performance was maintained (Figure 6d,e).

Different from mechanical reliability, temperature adaptiveness, such as antifreezing property, is another important aspect to evaluate device reliability. When the supercapacitor works at subzero temperature, its self‐healability would seriously deteriorate due to the freezing of hydrogel electrolyte. The supramolecular chemistry of host–guest interactions could effectively reduce this influence. Figure S22, Supporting Information, exhibited the electrochemical performance of assembled supercapacitor working at subzero temperatures. The CV profiles in Figure S22a, Supporting Information, displayed the close‐to‐rectangular shape even when the temperature decreased to −20 °C, which could be ascribed to good ionic conductivity of the supramolecular hydrogel electrolytes at low temperatures. In addition, compared to the freezing of PAA‐hydrogel‐based supercapacitor, the supramolecular supercapacitor cut in half could sufficiently heal to form one device at −20 °C, and the specific capacitance maintained over 85.2% retention after five cut/healing cycles, highly demonstrating the excellent self‐healing properties of polymer supercapacitor while working in cold conditions (Figure 6f,g).

To demonstrate the practical application, the supramolecular supercapacitor was utilized to drive a photodetector of perovskite nanowires (Figure 6h). Figure S23, Supporting Information, shows the circuit diagram of the assembled device, in which the photodetector was driven by an original and a healed supercapacitor, respectively. As shown in Figure 6i, the on/off photocurrent ratio of the circuit with healed supercapacitor only exhibited a slight decay than that with the original one, indicating the good restoration of the self‐healing polymer supercapacitor. These results highly demonstrate the excellent self‐healing properties and great potential of the self‐healing polymer supercapacitor in wearable electronic devices.

## 6 Conclusion

Traditional supercapacitors suffer from the generally overlooked issues of interlayer slippage, delamination, and crease formation while under deformations, which are all induced by the laminated multilayer configuration and inelastic component layers. To ultimately solve this problem, we should have a non‐laminated, all‐integrated structure with all components being elastic, including electrodes and electrolyte. As the hydrogel electrolyte is intrinsically elastic, the realization of fully elastic electrode is key to endow the whole device elasticity. After carefully studying the improving strategies from configuration and material perspectives, we design a highly reliable supercapacitor through an all‐elastic and all‐polymer approach. Specifically, regarding the electrolyte, the supramolecular hydrogel contains two kinds of *β*‐CD‐based host–guest inclusion complexes with superior self‐healing properties. On the other side, the PPy electrode is endowed with elasticity after been integrated into the elastic hydrogel electrolyte through extensive hydrogen bonding and covalent interactions with aromatic amino acid residues (Lys and Tyr) of silk fibroin chains. Of note, these abundant and strong interfacial bindings result in stable chemical adhesion between the contiguous electrode and electrolyte layers. Finally, the as‐obtained supramolecular supercapacitor is accommodated with targeted mechanical, structural, and physicochemical properties like high elasticity, non‐laminated configuration, self‐healing, and antifreezing capabilities. This protocol of fabricating the energy storage devices with all‐in‐one configuration is facile and effective, which would show great compatibility with other typical flexible structures such as the fiber‐shaped supercapacitor.^[^ ^33^, ^34^ ^]^

Subsequently, supercapacitors performance is systematically investigated from electrochemical and mechanical aspects. First, it delivered an excellent areal capacitance of 0.37 F cm^−2^ and a volumetric energy density of 0.082 mW h cm^−3^ in undeformed states. Second, thanks to the non‐laminated configuration and all‐components elastic design, the mechanical stability of the whole device is highly improved to endure various mechanical stimuli, avoiding the unexpected mechanical dysfunctionalities. Regarding the durability of the whole device subjected to even severer mechanical damages, exceptional self‐healing property of the non‐laminated configuration is realized by applying the dynamic supramolecular chemistry of host–guest interactions between the *β*‐CD molecules and amino acids of silk fibroin. It should be emphasized upon the self‐healability of electrode layer that it endows the healing capability at device level, where the generally applied electrodes are hard to recover. The intrinsic healing efficiency of supercapacitor approaches ≈95.8% even after the 30 cutting/healing cycles, which is comparable to other reported self‐healable supercapacitors with different healing mechanism or the devices using additional outer healing component. Furthermore, the supercapacitor retains over 85.2% retention of the specific capacitance after five cut/healing cycles even working at −20 °C. To the best of our knowledge, this is the first demonstration of the supramolecular supercapacitor with such extreme self‐healability and freeze‐resistance on comparing with substantial past research. Overall, this facile and effective assembly protocol for non‐laminated device configuration can effectively guarantee mechanical reliability of flexible supercapacitors under realistic applications, ranging from general mechanical manipulations to severe damages, which is expected to shed light on the future wide‐scale adoption of highly reliable supercapacitors.

## Conflict of Interest

The authors declare no conflict of interest.

## Supporting information

Supporting Information

## Data Availability

Research data are not shared.
