# Supplementary material for: A Self‐Healing Crease‐Free Supramolecular All‐Polymer Supercapacitor
Source: Adv Sci (Weinh). 2021 May 1;8(12):2100072. doi: 10.1002/advs.202100072 (PMC8224449; doi:10.1002/advs.202100072)
Supplement: Supplementary file 1 — Supporting Information [file ADVS-8-2100072-s001.pdf]

© 2021 Wiley-VCH GmbH

ADVANCED SCIENCE
Open Access

Supporting Information

for *Adv. Sci.,* DOI: 10.1002/advs.202100072

A self-healing Crease-free Supramolecular All-polymer Supercapacitor

Funian Mo, Qing Li, Guojin Liang, Yuwei Zhao, Donghong Wang, Yan Huang, Jun Wei,* and Chunyi Zhi**

Supporting Information

A self-healing crease-free supramolecular all-polymer supercapacitor

Funian Mo, Qing Li, Guojin Liang, Yuwei Zhao, Donghong Wang, Yan Huang, Jun Wei,* and Chunyi Zhi**

Methods

Preparation of Regenerated Silk Fibroin (SF).

The regenerated SF was prepared according to previous report.[1] Briefly, the raw silk (produced in Zhejiang, China) was first boiled in 0.05 wt % Na_2CO_3 (AR, Aladdin) for 90 min at 90 °C, and then thoroughly rinsed with deionized water and dried overnight at room temperature. Second, the degummed silk was dissolved in a solution containing $CaCl_2$ (AR, Aladdin), CH_3CH_2OH (AR, Aladdin) and H_2O (1:2:8) at 60 °C for 2 h and subsequently centrifuged at 8000 rpm for 12 min to remove aggregates. After that, the supernatant was continuously dialyzed in deionized water by using a cellulose dialysis membrane (MWCO 12400 Da) over 3 days to remove residual salts and then lyophilized to finally achieve the regenerated SF.

Preparation of SF Conjugated with *β*-Cyclodextrin (SF-CD).

The SF-CD was obtained through Schiff base formation.[2] 2 g *β*-CD (99.999%, Adamas) was dissolved in 50 mL DMSO (AR, Aladdin), then adding 2 Equiv of Dess-Martin periodinane (DMP, Energy Chemical). The reaction mixture was magnetically stirred for 1 h at room temperature. After that, addition of 300 mL acetone and cooling at -10 °C facilitated the isolation of the crude product *β*-CD monoaldehyde (*β*-CD-CHO) followed by filtration. The purification of *β*-CD-CHO was executed by repeating the dissolution of *β*-CD-CHO in

DMSO and the precipitation with acetone to remove the periodinane byproduct. The acetone and DMSO was removed by dissolving *β*-CD-CHO in water, followed by stirring for 1 h and lyophilization. The as-prepared *β*-CD-CHO was dissolved and incubated in a given volume of regenerated SF solutions at 25 °C for 24 h to finally yield SF-CD.

Preparation of *β*-CD Conjugated Acrylic Acid (AA-*β*-CD).

AA-*β*-CD was synthesized through grafting *β*-CD to acrylic acid (AA) side chains. 4 ml AA (Aladdin) was first dissolved in 20 ml N,N-dimethylformamide (DMF). Secondly, Benzotriazol-1-yl-oxytripyrrolidinophosphonium hexafluorophosphate (PyBOP, 0.06 eq. of AA unit) and Triethylamine (Et3N, 0.06 eq. of AA unit) were added into this solution. After magnetic stirring for 2 h, 2 g *β*-CD was added into the solution and further stirred for 12 h. The resultant product was then reprecipitated and washed with 200 ml ethanol. At last, the precipitate was dialyzed for 2 days with a dialytic tube, and lyophilized for 24 h to finally obtain the AA-*β*-CD.

Preparation of Supramolecular Hydrogels.

The designed supramolecular hydrogel was fabricated as follows: 10 mL 20 wt% SF-CD solution was first prepared with 0.5 M NaCl aqueous solution as solvent. 1 g PAA-*β*-CD, 0.035 g Ammonium persulphate (APS, AR, Aladdin), 0.005 mg N,N'-methylenebisacrylamide (AR, Aladdin) and 4 μL 20 mM $[Ru(bpy)_3]^{2+}Cl^{2-}$ (Aladdin) solution were sequentially added and mixed with the above aqueous solution by vortexing. Subsequently, the hybrid solution was injected into a mold and radiated under UV radiation (360 nm) for 30 min. Finally, the hydrogels were washed thoroughly with deionized water to

remove remaining reagents. For control group, PAA hydrogel was synthesized by replacing the dispersed monomer solution with equivalent acrylic acid.

In-situ Polymerization of PPy.

A piece of film materials (supramolecular hydrogel, common paper or cloth) with a typical area was soaked in 0.1-0.5 M pyrrole solution for 30 minutes. Subsequently, the films were transferred to 100 ml hybrid aqueous solution containing 8 g ferric chloride and 0.3 M hydrochloric acid, and kept at 4 °C for different times of 5, 30, 60 and 120 minutes. The films was then taken out and thoroughly washed with 0.3 M hydrochloric acid and deionized water to remove the unreacted regents. The resultant films were finally immersed into sodium silicate solution (AR, Sigma-Aldrich) for 30 min and washed with deionized water.

Materials Characterization

Tensile tests of hydrogels were conducted by an HTSLLY9130A tensile machine (Guangdong Zhongye Instrument Equipment Co., Ltd.). Prior to each test, the hydrogel samples were cut into the dumbbell shapes with 30 mm length and 10 mm width. The tensile modulus was determined by the average slope of the strain range of 20%-60% from stress-strain curve. Fourier transformation infrared (FT-IR) spectra of hydrogels were achieved with an FT-IR spectrometer (Nicolet 6700, Nicolet, USA) with 24 scans at a 4 cm^{-1} resolution signal averaged. The surface morphology of PPy/hydrogel was characterized by utilizing an environmental scanning electron microscope (ESEM, FEI/Philips XL30).

Electrochemical Measurements

To test the electrochemical performance, The PPy/supramolecular hydrogel film was cut into a sample (1 cm width × 3 cm length × 2 mm thickness). Cyclic voltammetry (CV) and galvanoststic charge/discharge (GCD) measurements of the supercapacitors were executed using an electrochemical workstation (CHI 760D). Electrochemical impedance spectroscopy (EIS) test was conducted in the frequency range of 0.01-10^5 Hz with a perturbation of 10 mV at open circuit voltage. Cycling performance test was performed by utilizing a Land 2001A battery testing system. The specific capacitances of supercapacitors were calculated from GCD curves under various current densities according to the equations below:

$$C_A = \frac{I \times \Delta t}{A \times \Delta V} \quad (1)$$

$$C_V = \frac{I \times \Delta t}{V \times \Delta V} \quad (2)$$

where C_A (mF cm^{-2}) is the areal specific capacitance, C_V (mF cm^{-3}) is the volumetric specific capacitance, I (mA) is the discharge current, Δt (s) is the discharge time, A (cm^{-2}) is the effective area of electrodes, i.e., the contact area between one electrode and the electrolyte, V (cm^{-3}) is the total volume of the whole device, and ΔV (V) is the operating voltage which determined from the discharge curves excluding potential drop.

The volumetric energy densities and power densities were calculated according to the following equations:

$$E_V = \frac{C_V \times \Delta V^2}{2 \times 3600} \quad (3)$$

$$P_V = \frac{E_V}{t} \quad (4)$$

where E_V (mWh cm^{-3}) is the volumetric energy density, C_V (mF cm^{-3}) is the volumetric specific capacitance, ΔV (V) is the operating voltage excluding potential drop, P_V (mW cm^{-3}) is the volumetric power density, t (h) is discharging time.

Preparation and Characterization of the Photodetector Driven by the Supercapacitor

First, the interdigitated electrodes were prepared on a doped Si substrate with a 300 nm SiO_2 dielectric layer through the methods of standard photolithography and electron beam evaporation (SYSKEY) of 6 nm Ti and 60 nm Au layers. After the lift-off process, a patterned substrates seven pairs of electrode fingers with 0.55 cm length and 15 μm gap spacing was obtained and cleaned in acetone, isopropanol, ethanol, and deionized water for 15 min. After that, the electrode was dried under a nitrogen gas flow, and subsequently treated with UV-ozone for 15 min to produce a hydrophilic surface. Second, 55 μL of perovskite precursor solution was spincoated at a speed of 5000 rpm for 60 s. Likewise, after 7 s of the spin stage, 100 μL of toluene acting as an antisolvent was quickly added. The time-dependent current characteristics were recorded on a source meter (Keithley 2450) with a three probe station with the supercapacitor serving as the external power supply. Monochromatic visible light at 525 nm was produced by laser flashlights, and infrared light was produced by an 880 nm pulsed laser diode.

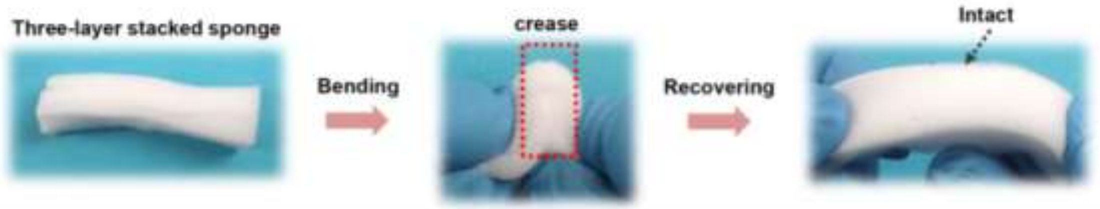

Figure S1. Schematic illustration of the three-layer elastic sponge upon bending deformation, which indicating the entire deformation of whole device without delamination due to all elastic components.

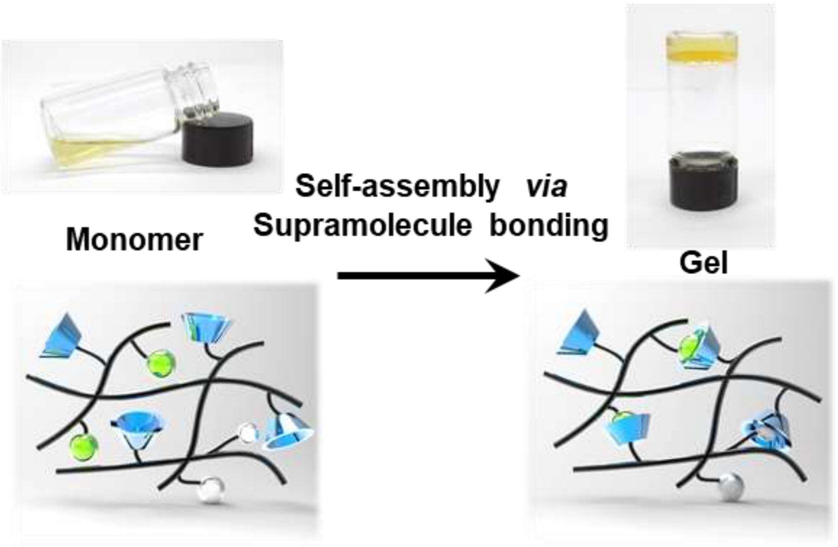

Figure S2. Formation of the supramolecular hydrogels..

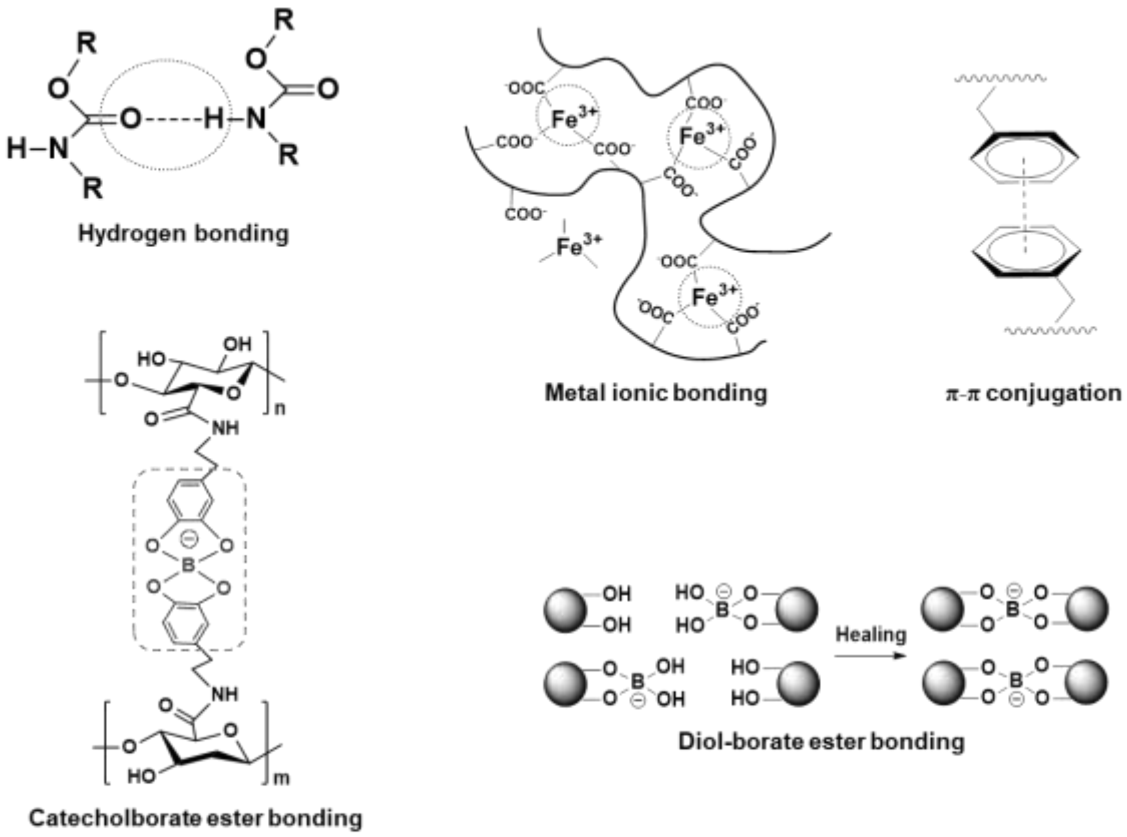

Figure S3. Chemical structures of traditional self-healable polymers based on diverse self-healing mechanism.[3-6]

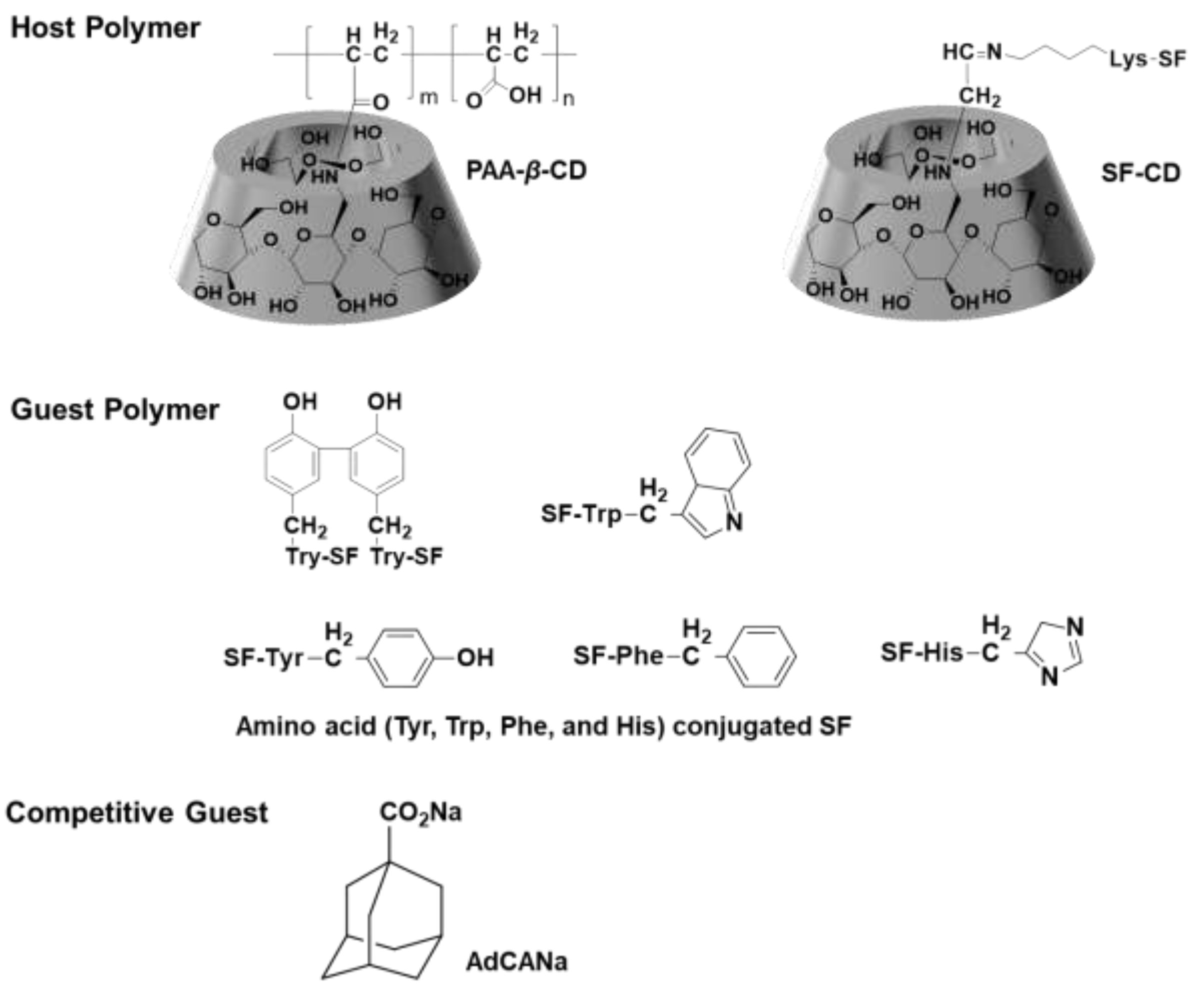

Figure S4. Chemical structures of the host polymers and guest polymers. Host polymers (PAA-*β*-CD and SF-CD) were prepared by side-chain modification of PAA and SF chains.

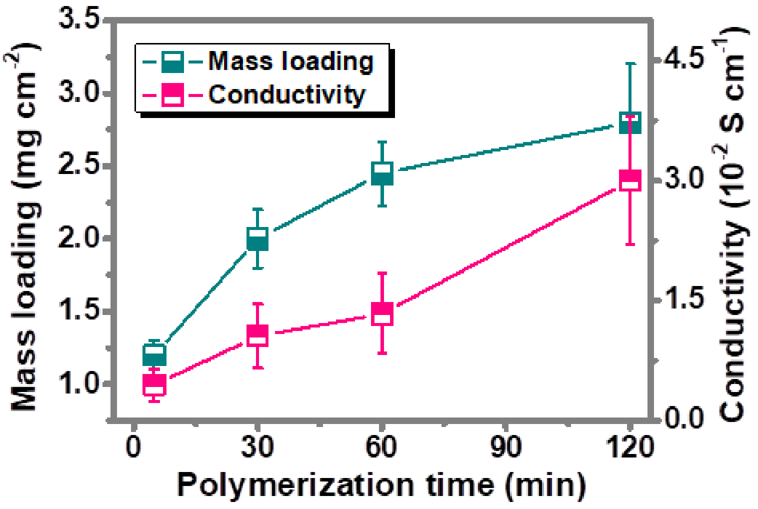

Figure S5. Mass loadings of the polymerized PPy and conductivity of the fabricated PPy/supramolecular hydrogel with respect to polymerization times of 5, 30, 60 and 120 minutes.

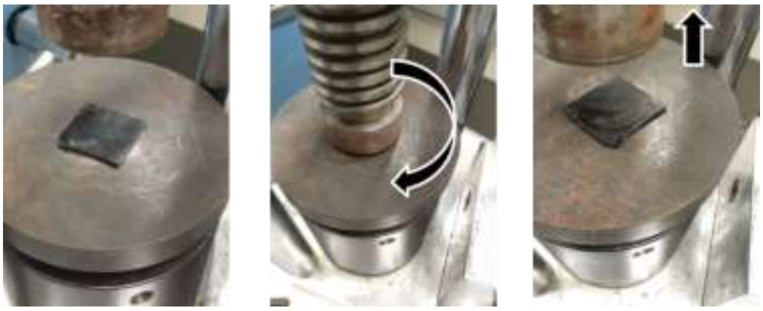

Figure S6. Mechanical adhesion test of the PPy and supramolecular hydrogel after helical compression.

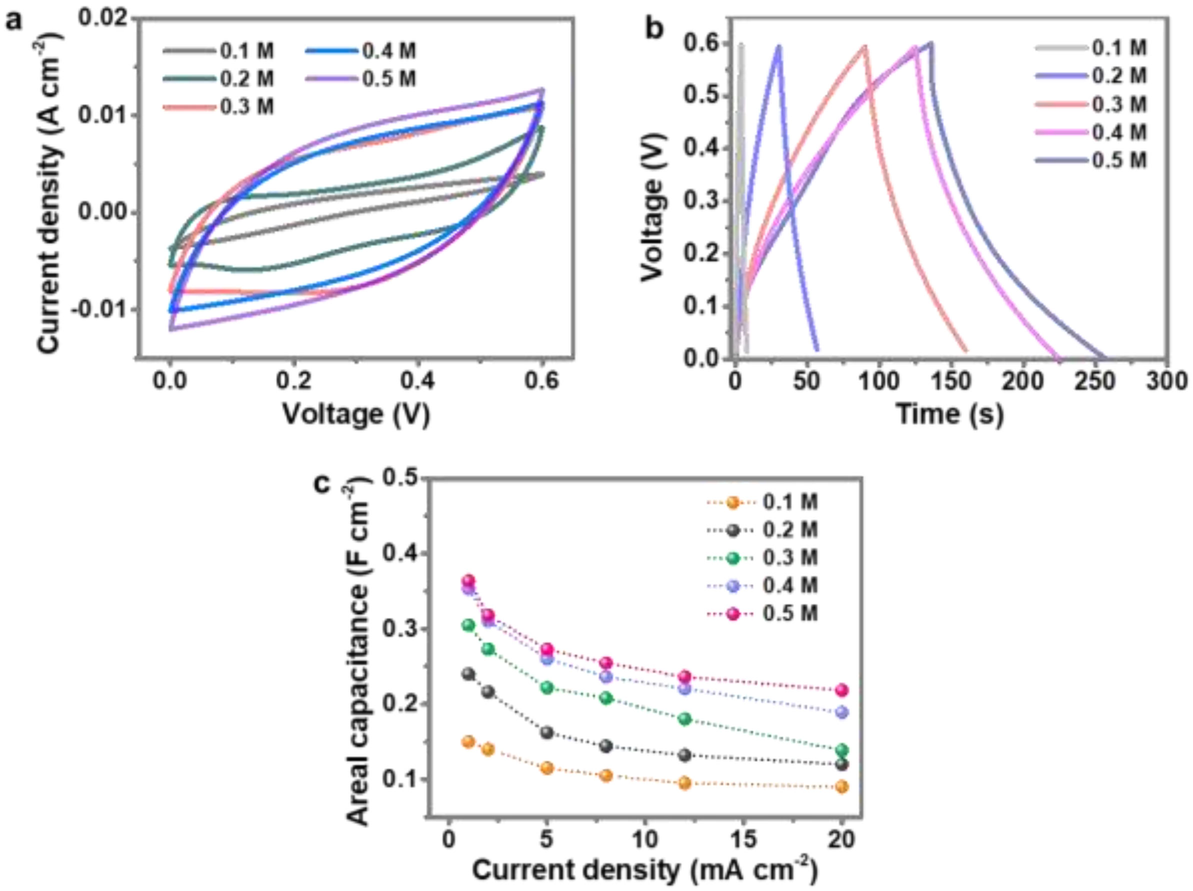

Figure S7. (a) CV curves of the supramolecular supercapacitor with various Py concentration at 50 mV s^{-1}. (b) GCD curves of the supramolecular supercapacitor with various Py concentration at 2 mA cm^{-2}. (c) Areal capacitance with respect to the discharge current of the supramolecular supercapacitor with various Py concentration.

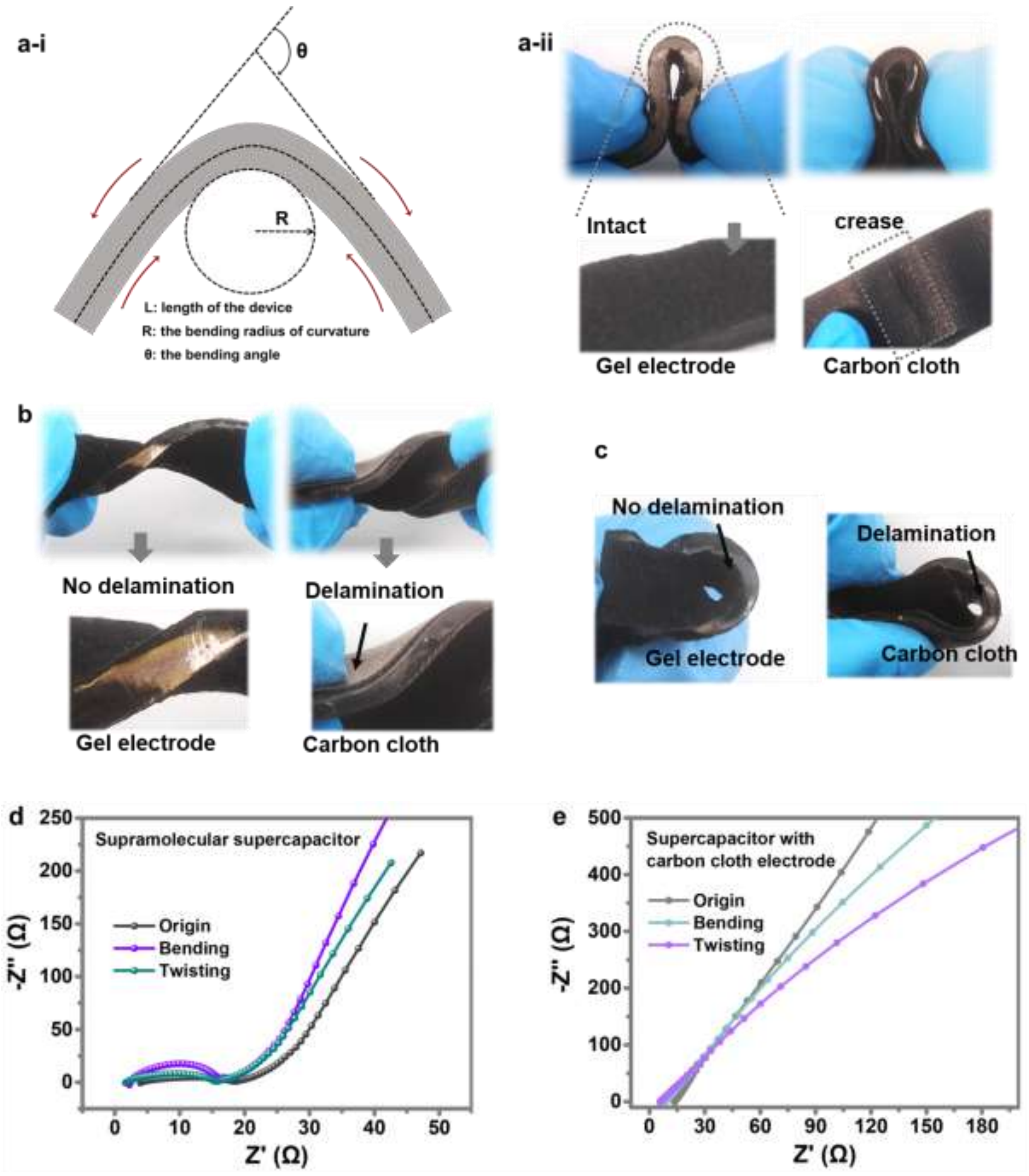

Figure S8. Magnified photographs showing the interfacial contact of the supramolecular supercapacitor and comparative supercapacitor with carbon cloth electrodes under (a) bending (L is length of the device, θ is the bending angle, R is the bending radius of curvature), (b) twisting, and (c) multiple deformations. Electrochemical impedance spectrums

of the (d) all-elastic supramolecular supercapacitor and (e) laminated supercapacitor with carbon cloth electrodes under different deformation states.

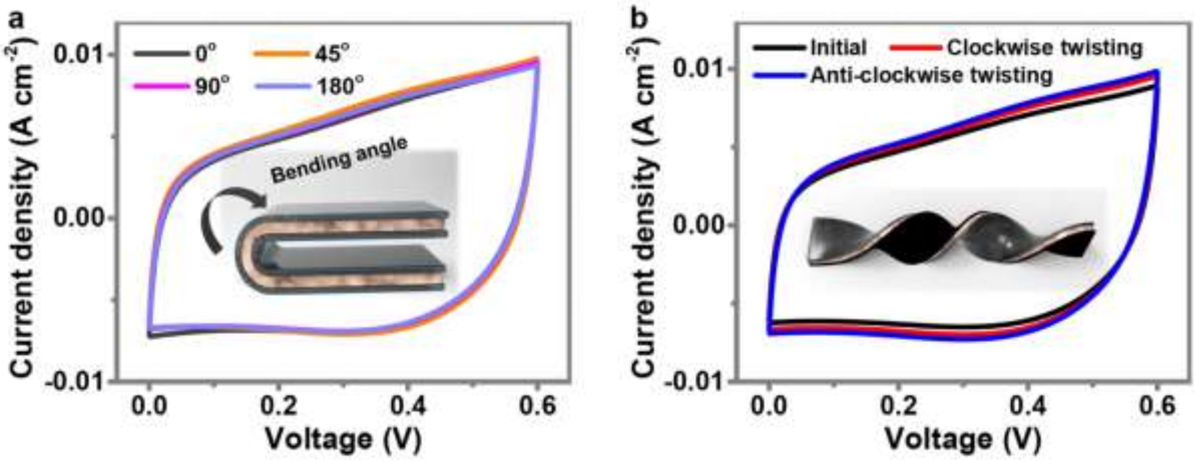

Figure S9. (a) CV curves of the supramolecular supercapacitor at various bending angles tested at the scan rate of 30 mV s^{-1}. (b) CV curves of the supramolecular supercapacitor at 30 mV s^{-1} while it being dynamically twisted.

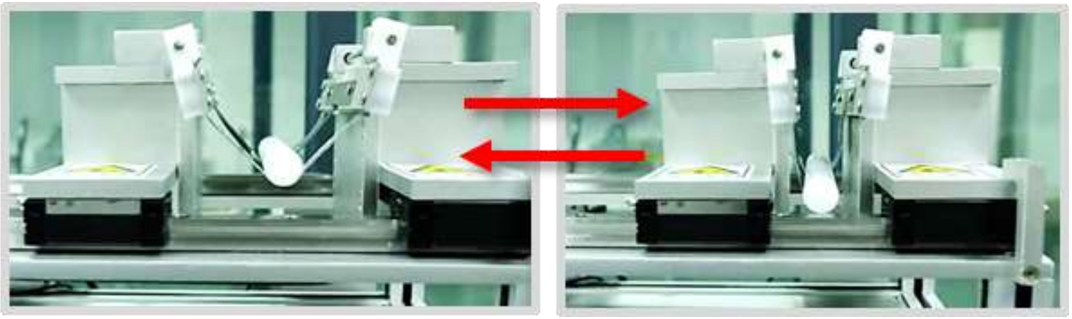

Figure S10. Digital photographs of the supramolecular supercapacitor in bending test.

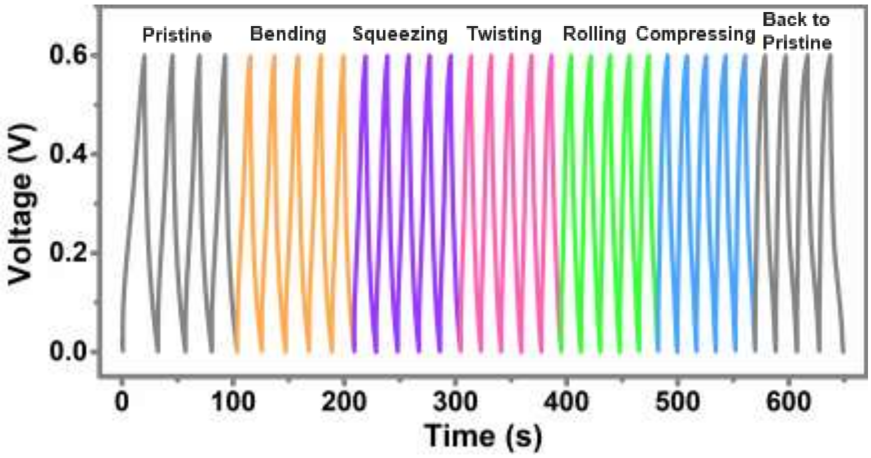

Figure S11. GCD profiles at 2 mA cm^{-2} of the supramolecular supercapacitor under various dynamical stimuli.

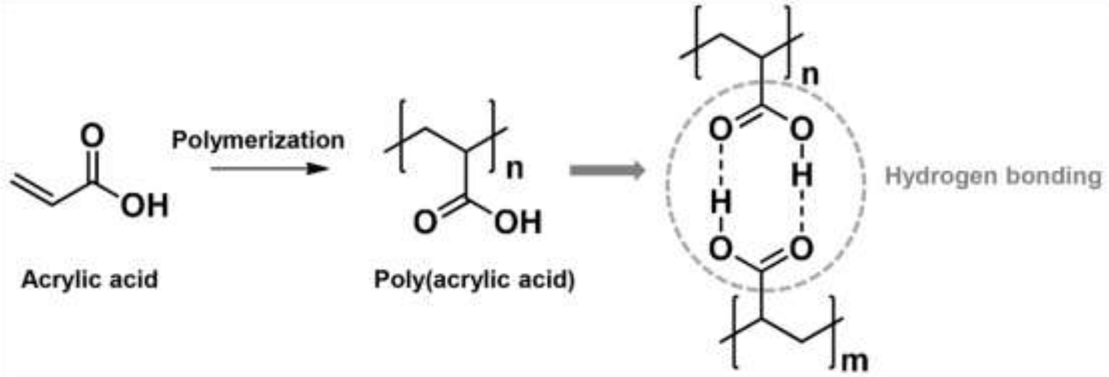

Figure S12. The synthesis routine and molecular structure of PAA hydrogel.

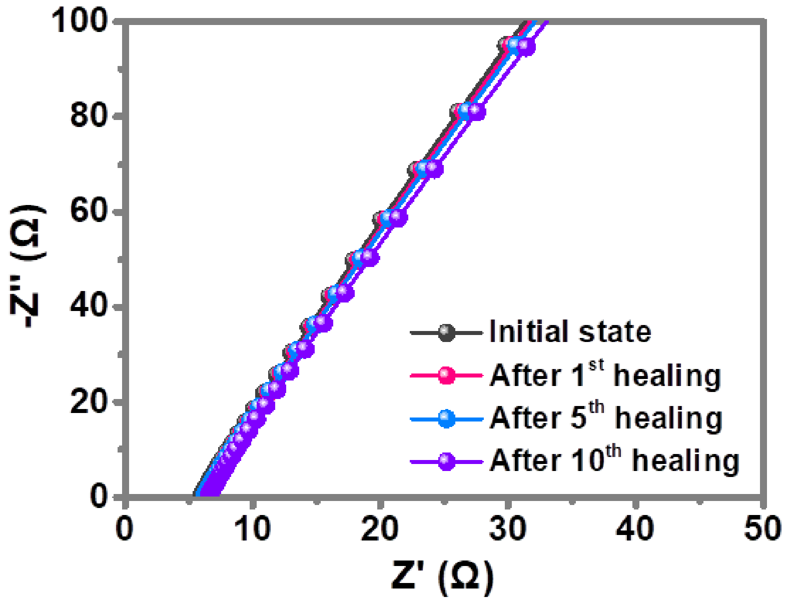

Figure S13. AC impedance spectra of the supramolecular hydrogel after multiple cutting-healing times.

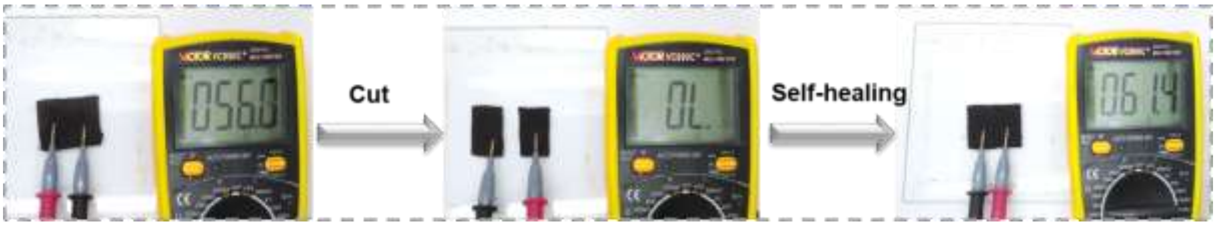

Figure S14. Optical observation and resistivity measurements of the PPy/supramolecular hydrogel in the self-healing process.

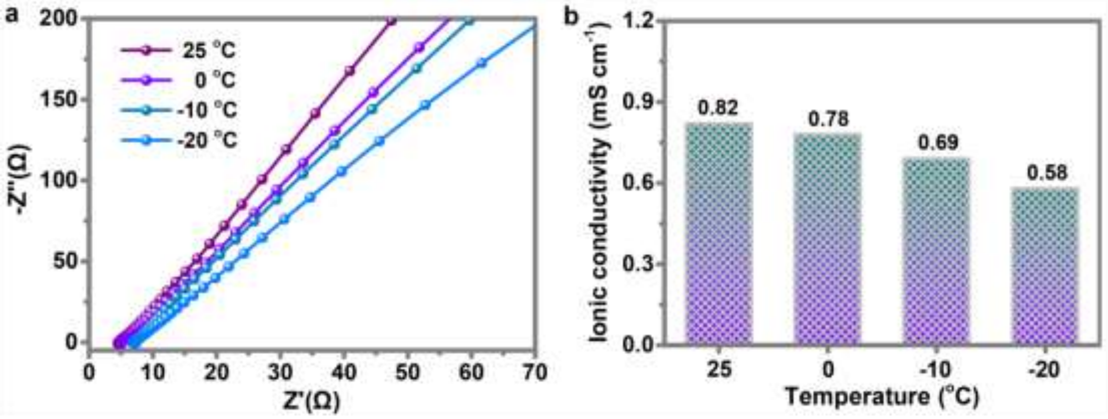

Figure S15. (a) AC impedance spectra of the supramolecular hydrogel electrolyte measured at different temperatures. (b) The calculated ionic conductivity of the supramolecular hydrogel electrolyte at different temperatures.

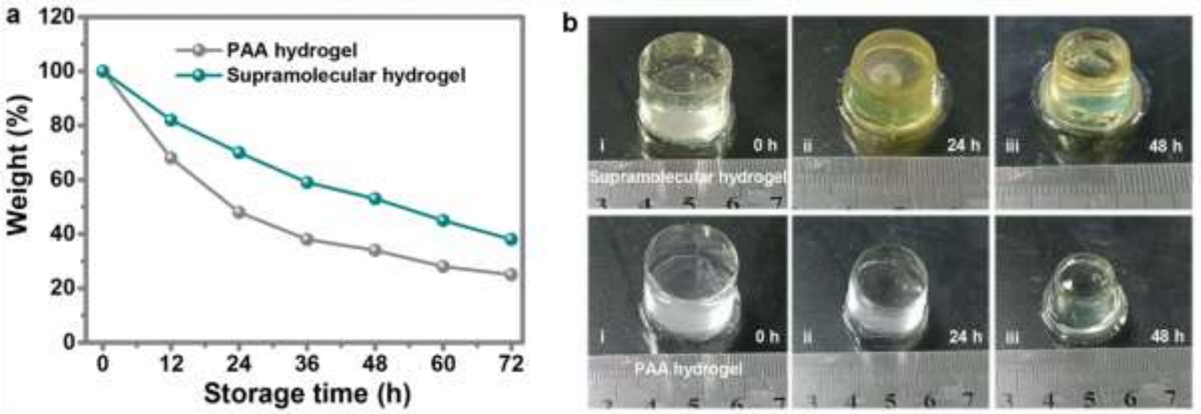

Figure S16. (a) A statistical graph and (b) the digital photos of the weight retention of the ZSC-gel and PAM-gel electrolytes after being storage in the open air for various days. The test was performed at 25 °C and 50% humidity, and the weight retention was recorded at different time intervals.

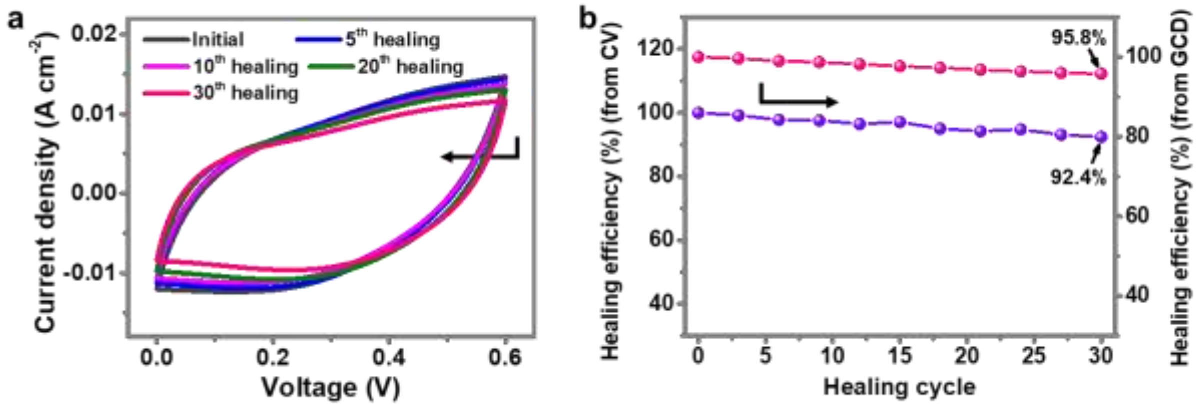

Figure S17. (a) CVcurves of the healed supercapacitor being performed during cutting–healing cycles. (b) Healing efficiency of the supramolecualr supercapacitor during various healing cycles.

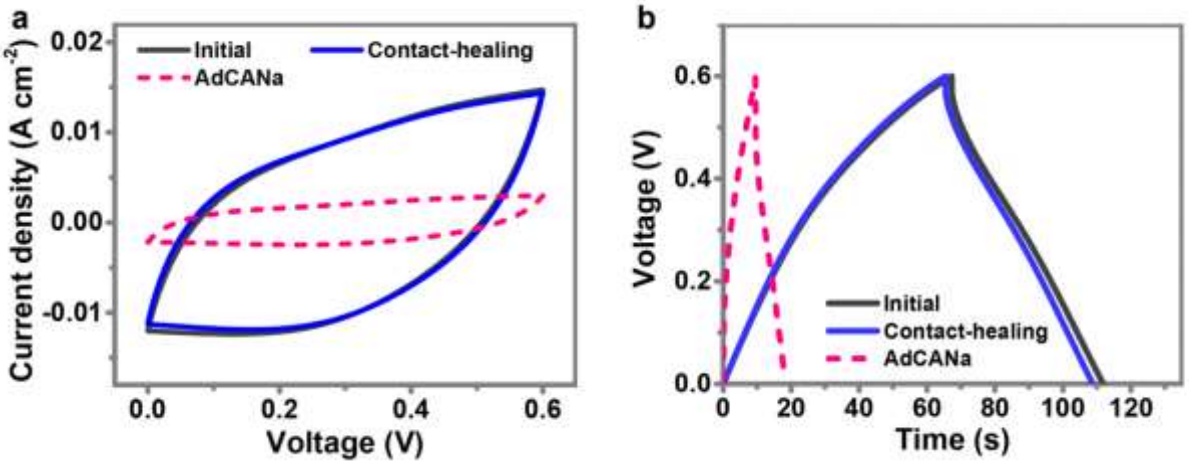

Figure S18. Comparisons of (a) CV curves and (b) GCD curves of the supramolecular supercapacitors with and without the competitive guest of AdCANa spread on the cut surface.

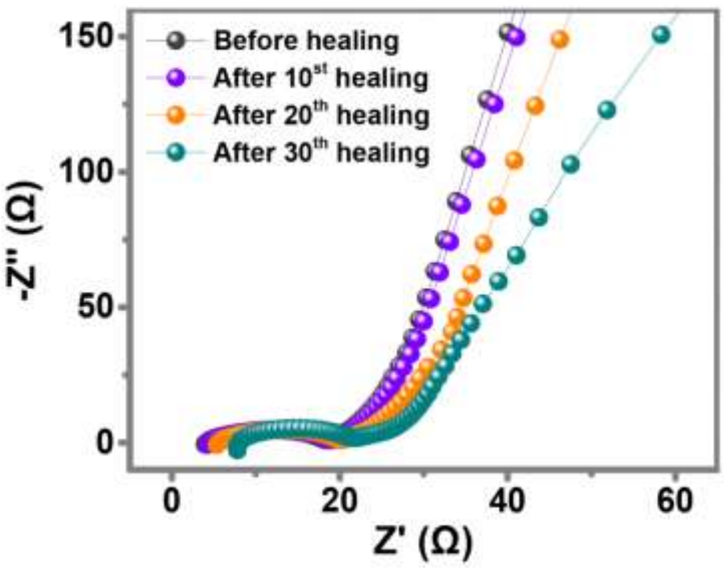

Figure S19. Electrochemical impedance spectroscopy (EIS) of the healed supercapacitor being performed during cutting–healing cycles.

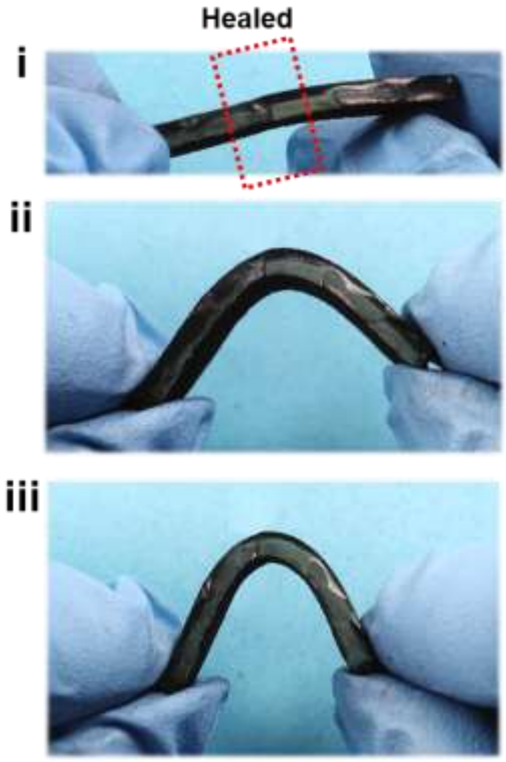

Figure S20. Electrochemical impedance spectroscopy (EIS) of the healed supercapacitor being performed during cutting–healing cycles.

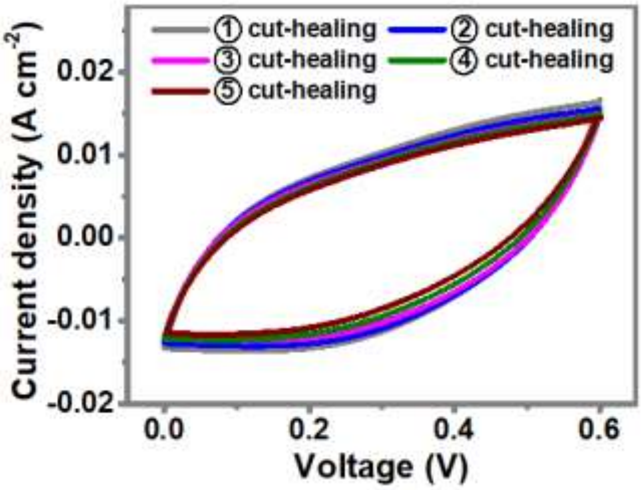

Figure S21. CVcurves of the healed supercapacitor during five cutting–healing process.

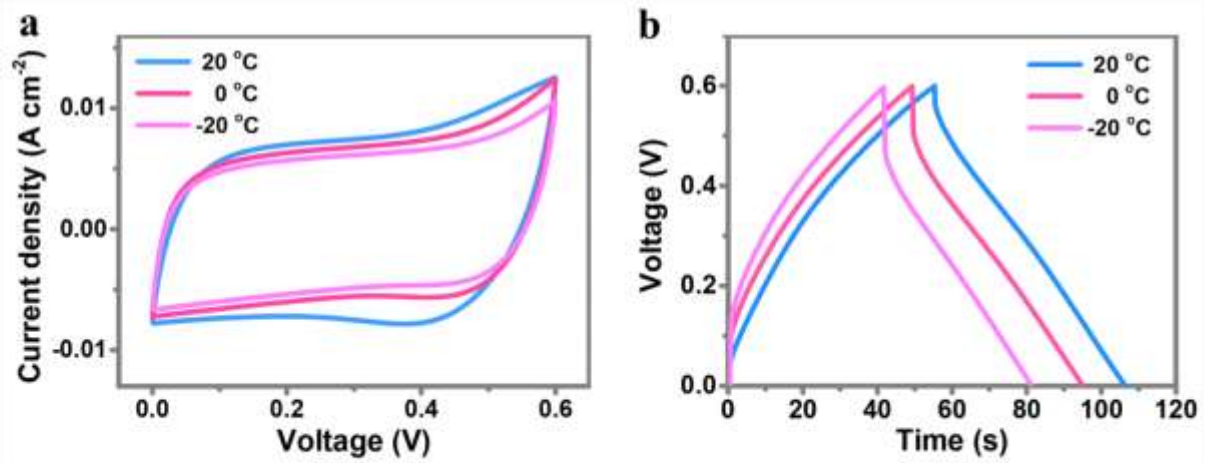

Figure S22. (a) CV curves of the supramolecular hydrogel electrolyte at low temperatures measured at 20 mV s^{-1}. (b) GCD curves of the supramolecular hydrogel electrolyte at low temperatures measured at 5 mA cm^{-2}

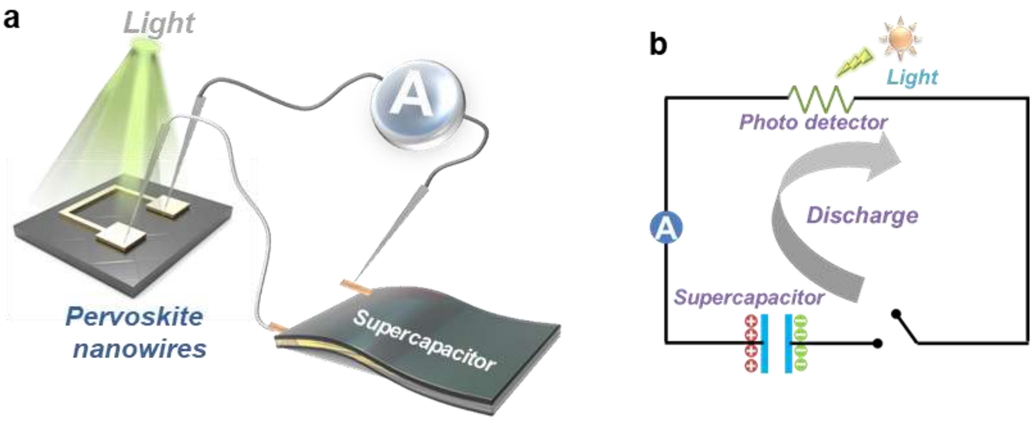

Figure S23. (a) Illustration and (b) circuit diagram of the perovskite nanowires based photodetector driven by the supercapacitor.

Table S1. Comparison of the presented supercapacitor with previously-reported self-healable supercapacitors in terms of healing mechanism and self-healing performance.

Ref.	Healable layer	Healable material	Healing mechanism	Maximum healing times	Last healing efficiency (%)	Healing at subzero temperatures
**This study**	**Omni**	**Supramolecular hydrogel**	**Host-guest interactions**	**30**	**95.8**	✓
[3]	Outer shell	Carboxylated PU	Hydrogen bonding	3	54.2	×
[7]	Outer shell	Healable PU	Hydrogen bonding	4	71.8	×
[8]	Electrode	Liquid-metal/ PU composites	Microcapsules release-Hydrogen bonding	5	75	×
[9]	Electrode	Extra substrate	Self-adhesion	5	85.7	×
[10]	Electrolyte	MGO-PAA	Hydrogen bonding	3	80	×
[11]	Electrolyte	PVA-H_2SO_4	Hydrogen bonding	5	80	×
[12]	Electrolyte	Fe^{3+}-PANa	Metal ionic bonding	4	87	×
[5]	Electrolyte	SA-g-DA/KCl	Catecholborate ester bonding	5	83	×
[6]	Electrolyte	PVA-g-PAA/KCl	Diol-borate ester bonding	16	78	×

References

[1] D. Zhang, H. Peng, B. Sun, S. Lyu, *Fibers Polym.* **2017**, *18*, 1831.

[2] Z. Youming, D. Xinrong, W. Liangcheng, W. Taibao, *J. Inclusion Phenom.Macrocyclic Chem.* **2008**, *60*, 313.

[3] S. Wang, N. Liu, J. Su, L. Li, F. Long, Z. Zou, X. Jiang, Y. Gao, *ACS Nano* **2017**, *11*, 2066.

[4] Y. Guo, X. Zhou, Q. Tang, H. Bao, G. Wang, P. Saha, *J. Mater. Chem. A* **2016**, *4*, 8769.

[5] F. Tao, L. Qin, Z. Wang, Q. Pan, *ACS Appl. Mat. Interfaces* **2017**, *9*, 15541.

[6] Z. Wang, F. Tao, Q. Pan, *J. Mater. Chem. A* **2016**, *4*, 17732.

[7] Y. Huang, Y. Huang, M. Zhu, W. Meng, Z. Pei, C. Liu, H. Hu, C. Zhi, *ACS nano* **2015**, *9*, 6242.

[8] S. Park, G. Thangavel, K. Parida, S. Li, P. S. Lee, *Adv. Mater.* **2019**, *31*, 1805536.

[9] H. Wang, B. Zhu, W. Jiang, Y. Yang, W. R. Leow, H. Wang, X. Chen, *Adv. Mater.* **2014**, *26*, 3638.

[10] X. Jin, G. Sun, H. Yang, G. Zhang, Y. Xiao, J. Gao, Z. Zhang, L. Qu, *J. Mater. Chem. A* **2018**, *6*, 19463.

[11] Y. Guo, K. Zheng, P. Wan, *Small* **2018**, *14*, 1704497.

[12] Y. Huang, J. Liu, J. Wang, M. Hu, F. Mo, G. Liang, C. Zhi, *Angew. Chem. Int. Ed.* **2018**, *57*, 9810.
